# Reply to: Cold induction of nuclear FRIGIDA condensation in *Arabidopsis*

**DOI:** 10.1038/s41586-023-06190-6

**Published:** 2023-07-12

**Authors:** Pan Zhu, Caroline Dean

**Affiliations:** John Innes Centre, Norwich Research Park, Norwich, UK

**Keywords:** Transcription, Plant development

replying to: Z. Zhang et al. *Nature* 10.1038/s41586-023-06189-z (2023)

We write in response to Zhang et al.^1^. Using their mutant we confirm our original findings. Zhang et al.^1^ analyse both F_1_ populations and *FRI-GFP* homozygous lines^2^ (but using different criteria for each) and report that “the cold-induced formation of nuclear FRI condensates is independent of *COOLAIR*”. Zhang et al.^1^ also find that “*COOLAIR* is not involved in *FLC* repression by prolonged cold exposure” because “loss of *COOLAIR* expression in either *FRIΔCOOLAIR-1* or *FRIΔCOOLAIR-2* had no effect on the progression of transcriptional shutdown of *FLC* during cold exposure or on post-cold stable silencing of *FLC*”.

**Fig. 1:**
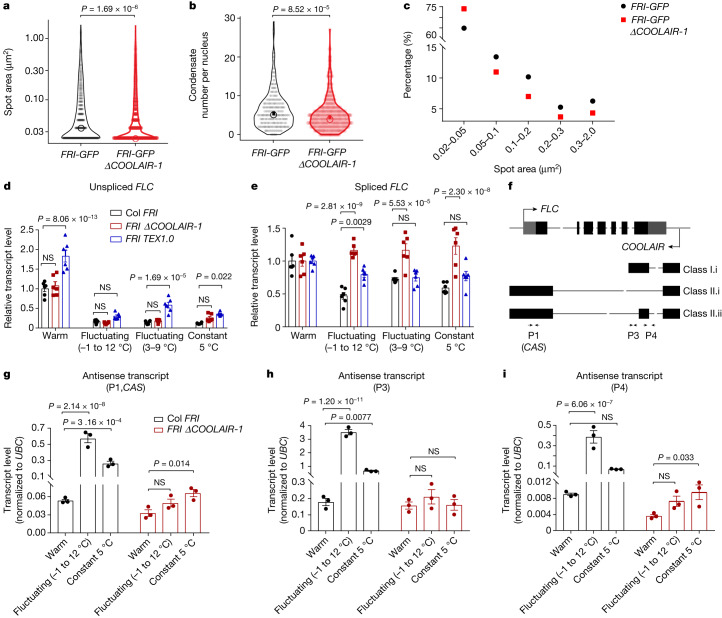
Cold-induced FRI–GFP condensate formation and *FLC* repression are attenuated in *ΔCOOLAIR-1*. **a**–**c**, Quantification of the FRI–GFP nuclear condensate area (spot area (**a**) and percentage (**c**)) and number per nucleus (**b**) in the roots of *FRI-GFP* and *FRI-GFPΔCOOLAIR-1* homozygous F_3_ lines. Plants were exposed to cold treatment for 4 days at a constant temperature of 5 °C. For **a** and **b**, the open circles indicate the median of the data and the vertical bars indicate the 95% confidence interval determined by bootstrapping. *n* = 1,729 and 2,548 condensates (**a**) and *n* = 271 and 529 (**b**) nuclei in *n* = 15 and 26 roots, respectively. Individual data points are shown as black or red dots. Comparison of mean values was performed using two-tailed *t*-tests with Welch’s correction. **d**,**e**, The relative expression level of unspliced *FLC* (**d**) and spliced *FLC* (**e**) in Col *FRI*, *FRI ΔCOOLAIR-1* and *FRI* *TEX1.0* plants with 2 weeks of growth under the indicated temperature conditions^6^. Data are mean ± s.e.m. of *n* = 6 biologically independent experiments. **f**, Schematic of *FLC* and *COOLAIR* transcripts at the *FLC* locus. Untranslated regions are indicated by grey boxes and exons by black boxes. Head-to-head arrows indicate primers used for antisense transcript level analysis by quantitative PCR (qPCR). **g**–**i**, The relative expression level of antisense transcripts at *FLC*, including but not limited to *CAS*^6^, by the indicated primers (P1 (*CAS*) (**g**), P3 (**h**) and P4 (**i**)) in Col-*FRI* and *FRIΔCOOLAIR-1* plants with 24 h of growth under the indicated temperature conditions. The primers used are indicated in **f**. Data are mean ± s.e.m. of *n* = 3 biologically independent experiments. NS, not significant; the exact *P* values are shown at the top of each comparison. Source data

We have combined the He laboratory Δ*COOLAIR-1* deletion^3^ with our *FRI-GFP* transgene^2^ (*FRI-GFP* Δ*COOLAIR-1*) and repeated the analysis. In contrast to their data, we show that deletion of the *COOLAIR* promoter significantly attenuates cold-induced formation of FRI–GFP nuclear condensates, changing the overall size distribution^4^ (Fig. 1a–c and Extended Data Fig. 1a–c). This fully confirms our original findings that antisense transcription is one component of the multiple cold responsive factors regulating FRI condensates^2^. FRI–GFP condensates show concentration dependency and plasticity to environmental conditions, both well-known properties of condensate dynamics^5^.

We disrupted *COOLAIR* expression in our original report using a terminator exchange construct (*TEX1.0*)^6^ and, indeed, the level of FRI–GFP is reduced in this line as it is in the *frl1-1* mutant (discussed in our original paper), a component of the FRI complex^2^. These effects are not due to transgene-induced RNA silencing of *FRI-GFP* (Extended Data Fig. 1d). We also confirm that the repression of spliced *FLC* RNA levels, which is particularly sensitive to widely fluctuating cold conditions^6^, is attenuated in the *FRI ΔCOOLAIR-1* line (Fig. 1d,e). Over the years of study, we find that *FLC* downregulation in the cold, even in wild-type plants, is very dependent on growth conditions and seedling density, with growth being essential for RNA reduction. Cold-induced downregulation of *FLC* expression is mediated through several different mechanisms^7–13^ with *COOLAIR* affecting the dynamics of many of these, and not only through FRI condensation.

The *ΔCOOLAIR-1* line still produces abundantly expressed antisense transcripts^6^ (Extended Data Fig. 1e–k). The marked robustness of antisense/non-coding expression at loci such as *FLC* shows the intrinsic connection of sense/antisense transcription, as has been found at many yeast loci^14,15^.

These antisense transcripts are inducible by short cold exposure (Fig. 1f–i) but are indeed downregulated after longer exposure (Extended Data Fig. 1f,h,i). The *TEX1.0* line has different alternative antisense transcript levels compared with the *ΔCOOLAIR-1* line (Extended Data Fig. 1e–k). We chose to use the *TEX1.0* line in our original study because it has the lowest levels of alternative antisense transcripts^6^ (Extended Data Fig. 1e–k). The sequence of events during cold-induced *FLC* silencing is very dynamic and condition dependent due to the nonlinearity of *FLC* transcriptional shutdown and epigenetic silencing dynamics. This nonlinearity emerges from the complex feedback mechanisms interconnecting non-coding transcription, chromatin modifications and RNA stability.

## Methods

The reference genotype Col *FRI*^2^, the *FRI-GFP*^2^ transgenic plants, *TEX1.0*^2,6^ and *ΔCOOLAIR-1*^3^ have been described previously. *ΔCOOLAIR-1* was crossed with Col *FRI* and *FRI-GFP* to generate the *FRI ΔCOOLAIR-1* and *FRI-GFP ΔCOOLAIR-1* lines. Imaging and quantification of FRI–GFP condensates were performed as described previously^2^. The experiments under fluctuating conditions and all of the qPCR with reverse transcription analyses were performed as previously described^6^. A list of all of the primers used in the qPCR assay are provided in Extended Data Table 1.

## Reporting summary

Further information on research design is available in the Nature Portfolio Reporting Summary linked to this article.

## Online content

Any methods, additional references, Nature Portfolio reporting summaries, source data, extended data, supplementary information, acknowledgements, peer review information; details of author contributions and competing interests; and statements of data and code availability are available at 10.1038/s41586-023-06190-6.

### Supplementary information

Reporting Summary

### Source data

Source Data Fig. 1

**Extended Data Fig. 1:**
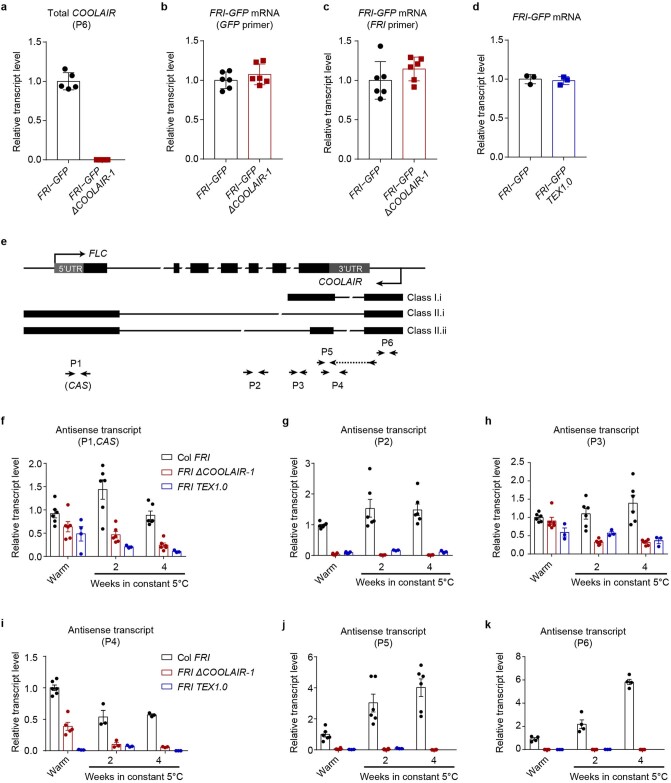
*FRI-GFP* and antisense transcript levels in *ΔCOOLAIR-1* and *TEX1.0*. **a**–**c**, Relative expression level of total *COOLAIR* and *FRI-GFP* mRNA assayed using the indicated primers in *FRI-GFP* and *FRI-GFP ΔCOOLAIR-1* F3 lines. Plants were given 4 days of cold treatment at constant 5 °C as in Fig. 1a–c. **d**, Relative expression level of *FRI-GFP* mRNA assayed in *FRI-GFP TEX1.0*. (**a**–**d**) Mean ± s.e.m.; n = 3 biologically independent experiments. **e**, Schematic of *FLC* and *COOLAIR* transcripts at the *FLC* locus. Untranslated regions are indicated by grey boxes and exons by black boxes. Head-to-head arrows indicate primers used for antisense transcript level analysis by qPCR. **f**–**k**, Relative expression level of antisense transcripts at *FLC* by the indicated primers in Col *FRI*, *FRI* *ΔCOOLAIR-1*, and *FRI* *TEX1.0* plants with the indicated lengths of cold treatment at constant 5 °C. The previously reported *CAS*^6^ was detected by P1 primer in (**f**). The primers used are indicated in (**e**). Mean ± s.e.m.; n = 3 or 6 biologically independent experiments as shown by the individual data points. Source data

Source Data Extended Data Fig. 1

## Figures and Tables

**Extended Data Table 1:**
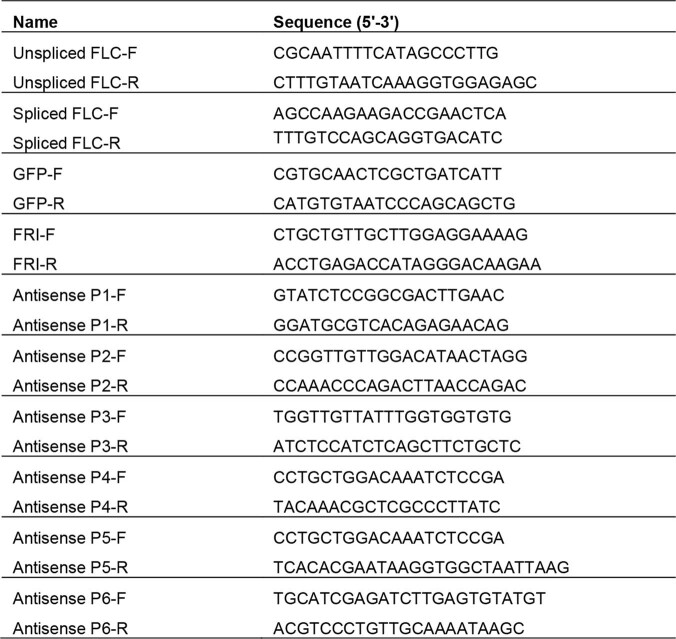
List of primers used in this study

## Data Availability

Source data are provided with this paper.
