# Supplementary material for: Reply to: Cold induction of nuclear FRIGIDA condensation in Arabidopsis
Source: Nature. 2023 Jul 12;619(7969):E33–7. doi: 10.1038/s41586-023-06190-6 (PMC10338334; doi:10.1038/s41586-023-06190-6)
Supplement: Supplementary file 1 — Reporting Summary [file 41586_2023_6190_MOESM1_ESM.pdf]

nature portfolio

Corresponding author(s): Caroline dean

Last updated by author(s): Mar 30, 2023

Reporting Summary

Nature Portfolio wishes to improve the reproducibility of the work that we publish. This form provides structure for consistency and transparency in reporting. For further information on Nature Portfolio policies, see our Editorial Policies and the Editorial Policy Checklist.

Statistics

For all statistical analyses, confirm that the following items are present in the figure legend, table legend, main text, or Methods section.

n/a	Confirmed	
☐	☒	The exact sample size (*n*) for each experimental group/condition, given as a discrete number and unit of measurement
☐	☒	A statement on whether measurements were taken from distinct samples or whether the same sample was measured repeatedly
☐	☒	The statistical test(s) used AND whether they are one- or two-sided<br>*Only common tests should be described solely by name; describe more complex techniques in the Methods section.*
☒	☐	A description of all covariates tested
☐	☒	A description of any assumptions or corrections, such as tests of normality and adjustment for multiple comparisons
☒	☐	A full description of the statistical parameters including central tendency (e.g. means) or other basic estimates (e.g. regression coefficient) AND variation (e.g. standard deviation) or associated estimates of uncertainty (e.g. confidence intervals)
☐	☒	For null hypothesis testing, the test statistic (e.g. *F*, *t*, *r*) with confidence intervals, effect sizes, degrees of freedom and *P* value noted<br>*Give P values as exact values whenever suitable.*
☒	☐	For Bayesian analysis, information on the choice of priors and Markov chain Monte Carlo settings
☒	☐	For hierarchical and complex designs, identification of the appropriate level for tests and full reporting of outcomes
☒	☐	Estimates of effect sizes (e.g. Cohen's *d*, Pearson's *r*), indicating how they were calculated

Our web collection on statistics for biologists contains articles on many of the points above.

Software and code

Policy information about availability of computer code

Data collection	All QPCR reactions were run with LightCycler® 480; all confocal images were collected on Zeiss LSM880 confocal microscope.
Data analysis	Microsoft Excel (version 2102, 64-bit), GraphPad Prism 7, Fiji-ImageJ 1.52i (Java 1.8.0_172, 64-bit)). Web service: PlotsOfData (https://huygens.science.uva.nl/PlotsOfData/).

For manuscripts utilizing custom algorithms or software that are central to the research but not yet described in published literature, software must be made available to editors and reviewers. We strongly encourage code deposition in a community repository (e.g. GitHub). See the Nature Portfolio guidelines for submitting code & software for further information.

Data

Policy information about availability of data

All manuscripts must include a data availability statement. This statement should provide the following information, where applicable:

- Accession codes, unique identifiers, or web links for publicly available datasets
- A description of any restrictions on data availability
- For clinical datasets or third party data, please ensure that the statement adheres to our policy

Source data are provided with this paper.

Field-specific reporting

Please select the one below that is the best fit for your research. If you are not sure, read the appropriate sections before making your selection.

☒ Life sciences ☐ Behavioural & social sciences ☐ Ecological, evolutionary & environmental sciences

For a reference copy of the document with all sections, see nature.com/documents/nr-reporting-summary-flat.pdf

Life sciences study design

All studies must disclose on these points even when the disclosure is negative.

Sample size	The sample size and the results of statistical analyses are described in the relevant figure legends. No statistical approach was used to predetermine sample size. Sample sizes were determined based on previous publications on similar experiments. The determined sample size was adequate as the differences between experimental groups was significant and reproducible.<br>RNA expression analysis (https://doi.org/10.1038/s41586-020-2485-4)<br>Microscopy and image quantification (https://doi.org/10.1038/s41586-019-1165-8; https://doi.org/10.1038/s41586-020-2485-4)
Data exclusions	No data was excluded from analysis.
Replication	All data contained at least three biologically independent replicates.
Randomization	Plants of different genotypes were grown side by side to minimize unexpected environmental variations during growth and experimentation. Different treatments were carried out in parallel, with minimum covarying factors. Seedlings at the same developmental stage were collected and assessed randomly for each genotype/treatment.
Blinding	Blinding was not applicable for this study because plants grown at different temperature conditions need to be collected at different growing time points and require specific handling temperatures.

Reporting for specific materials, systems and methods

We require information from authors about some types of materials, experimental systems and methods used in many studies. Here, indicate whether each material, system or method listed is relevant to your study. If you are not sure if a list item applies to your research, read the appropriate section before selecting a response.

Materials & experimental systems

n/a	Involved in the study	
☒	☐	Antibodies
☒	☐	Eukaryotic cell lines
☒	☐	Palaeontology and archaeology
☒	☐	Animals and other organisms
☒	☐	Human research participants
☒	☐	Clinical data
☒	☐	Dual use research of concern

Methods

n/a	Involved in the study	
☒	☐	ChIP-seq
☒	☐	Flow cytometry
☒	☐	MRI-based neuroimaging
